# Investigating cortical complexity and connectivity in rats with schizophrenia

**DOI:** 10.3389/fninf.2024.1392271

**Published:** 2024-08-15

**Authors:** Zongya Zhao, Yifan Feng, Menghan Wang, Jiarong Wei, Tao Tan, Ruijiao Li, Heshun Hu, Mengke Wang, Peiqi Chen, Xudong Gao, Yinping Wei, Chang Wang, Zhixian Gao, Wenshuai Jiang, Xuezhi Zhou, Mingcai Li, Chong Wang, Ting Pang, Yi Yu

**Affiliations:** ^1^School of Medical Engineering, Xinxiang Medical University, Xinxiang, China; ^2^Engineering Technology Research Center of Neurosense and Control of Henan Province, Xinxiang, China; ^3^Henan International Joint Laboratory of Neural Information Analysis and Drug Intelligent Design, Xinxiang, China; ^4^Center of Image and Signal Processing, Faculty of Computer Science and Information Technology, Universiti Malaya, Kuala Lumpur, Malaysia

**Keywords:** schizophrenia, electrocorticogram, brain network, complexity, connectivity

## Abstract

**Background:**

The above studies indicate that the SCZ animal model has abnormal gamma oscillations and abnormal functional coupling ability of brain regions at the cortical level. However, few researchers have focused on the correlation between brain complexity and connectivity at the cortical level. In order to provide a more accurate representation of brain activity, we studied the complexity of electrocorticogram (ECoG) signals and the information interaction between brain regions in schizophrenic rats, and explored the correlation between brain complexity and connectivity.

**Methods:**

We collected ECoG signal from SCZ rats. The frequency domain and time domain functional connectivity of SCZ rats were evaluated by magnitude square coherence and mutual information (MI). Permutation entropy (PE) and permutation Lempel-Ziv complexity (PLZC) were used to analyze the complexity of ECoG, and the relationship between them was evaluated. In addition, in order to further understand the causal structure of directional information flow among brain regions, we used phase transfer entropy (PTE) to analyze the effective connectivity of the brain.

**Results:**

Firstly, in the high gamma band, the complexity of brain regions in SCZ rats is higher than that in normal rats, and the neuronal activity is irregularity. Secondly, the information integration ability of SCZ rats decreased and the communication of brain network information was hindered at the cortical level. Finally, compared with normal rats, the causal relationship between brain regions of SCZ rats was closer, but the information interaction center was not clear.

**Conclusion:**

The above findings suggest that at the cortical level, complexity and connectivity are valid biomarkers for identifying SCZ. This bridges the gap between peak potentials and EEG. This may help to understand the pathophysiological mechanisms at the cortical level in schizophrenics.

## 1 Introduction

Schizophrenia (SCZ) is a commonly serious mental illness that severely impairs mental activity and social function of patients (Mccutcheon et al., 2020). Its lifetime prevalence rate is about 1%, causing a serious social burden. Therefore, the research and discovery of biomarkers of SCZ are clinically important for the early diagnosis of SCZ.

Electroencephalogram (EEG) is that postsynaptic potentials between brain cells discharge reflected electrophysiological activity on the scalp surface (van der Stelt and Belger, 2007). EEG has the advantages of high time resolution, low equipment price and abundant frequency band information. In recent years, EEG has been widely used to study brain cognitive function in patients with SCZ. Wang et al. (2023) used phase lag index (PLI) to construct brain networks that patients with SCZ showed reduced left frontal to posterior parietal/temporal connectivity compared to healthy control group. Krukow et al. (2018) used PLI to construct a brain functional network and observed that significantly higher PLI values were recorded in theta frequency, especially in the posterior areas and decreased PLI in low-alpha frequency within the frontal regions. Ibáñez-Molina et al. (2018) indicated that patients showed higher complexity values in right frontal regions only at rest, where no differences in complexity between patients and controls were found during the naming task. Yu et al. (2016) analyzed the fractal dimension (FD) values of EEG signals and found that EEG signals of patients with first-episode SCZ were more irregular and complex during the execution of functional tasks. However, EEG has a low spatial resolution and is sensitive to volume conduction effect, which cannot reflect the deep neural electrical activity of the brain (Cohen, 2017). Compared with EEG, electrocorticogram (ECoG) has higher spatial resolution, strong anti-noise ability, and can record stable high-frequency signals, which can accurately measure the activity of a certain part of the brain neurons (Donaldson et al., 2022).

ECoG is the sum of electrical signals generated by pyramidal cells on the surface of the cortex (Chatterjee et al., 2022). In clinical practice, ECoG is commonly used to accurately identify epileptic lesions (Piantoni et al., 2021), providing a new approach for studying human cortical activity (Jackson and Bolger, 2014). At present, ECoG is also applied to explore the pathophysiological mechanisms of intracortical and subcortical networks in patients with SCZ. Ahnaou et al. (2017) collected ECoG signals to evaluate the efficacy of drugs with potential antipsychotic properties and observed abnormal gamma oscillations in the animal model of SCZ, with network connectivity interrupted. Yan et al. (2022) collected ECoG signals from monkeys and found that high-gamma oscillations increased and low-band oscillations decreased in SCZ. Hashimoto et al. (2009) collected ECoG to study cortical auditory evoked responses in Nonhuman Primates of SCZ, and the results showed that the auditory evoked responses and latency were significantly increased in the animal model of SCZ. Komatsu and Ichinohe (2020) collected ECoG from the frontal region to analyze power changes in schizophrenic rats. The above studies indicate that the SCZ animal model has abnormal gamma oscillations and abnormal functional coupling ability of brain regions at the cortical level. However, few researchers have focused on the correlation between brain complexity and connectivity at the cortical level. In order to provide a more accurate representation of brain activity, we studied the complexity of ECoG signals and the information interaction between brain regions in schizophrenic rats, and explored the correlation between brain complexity and connectivity. Combining connectivity with complexity and applying it to neurophysiological data can provide new understanding of neural network processes in both healthy brains and pathological states.

The neural activity of the human brain as reflected by ECoG is a complex activity characterized by nonlinear dynamics (Ando et al., 2022). Therefore, the nonlinear correlation method is beneficial in helping us to understand and explain the ECoG kinetic features and the corresponding brain neural activity processes. Complexity and entropy are widely used in the study of nonlinear behavior of EEG signals. There has been an increasing trend toward the use of complexity analysis in quantifying neural activity measured by EEG signals (Ando et al., 2022). Xiang J. et al. (2019) used fuzzy entropy to explore brain neural activity and found that neural activity in the frontal and occipital regions of SCZ patients was more chaotic. Jia and Gu (2019) used sample entropy to characterize the nonlinear characteristics of the brain and observed irregular neural activity in the brain of patients with SCZ. SCZ is associated not only with localized functional deficits, but also with abnormal interactions between different brain regions. The analysis of functional brain connectivity in the resting state of patients with SCZ has attracted a great deal of attention. Functional connectivity reflects the integration of brain information processes in each neural region (Harmah et al., 2020), which is expressed as the interaction of neural activity between brain regions. Hummer et al. (2020) measured the Non-linear Directed Information Flow in SCZ by Multivariate Transfer Entropy and found that the interaction of neural activity in brain regions was weakened in patients with SCZ. Hummer et al. (2020) analyzed the functional connectivity of the whole brain in early-stage SCZ and found that the functional coupling between the networks of patients decreased. Fernández et al. (2013) proposed a functional disconnection syndrome in SCZ, suggesting that this disconnection is associated with higher complexity. However, the relationship between complexity and connectivity is unclear. Therefore, this paper uses a combination of complexity and connectivity to probe abnormal neuronal synchronization and abnormal neuronal firing in a rat model of SCZ.

The rapid discharge of PV+ interneurons produced gamma oscillations. However, the expression of PV positive intermediate neurons decreased in patients with SCZ (Jadi et al., 2016). The number of synapses of GABAergic interneurons is reduced in patients with schizophrenia, and the secretion and reabsorption of neurotransmitters by GABAergic interneurons are markedly impaired (Yuan et al., 2016). Therefore, patients with SCZ have abnormal gamma oscillations. And this reflects the fact that gamma oscillations are likely to be electrophysiologic markers of early schizophrenia. Brennan et al. (2018) found that abnormal gamma oscillations in SCZ are associated not only with perceptual or lower-order cognitive processing, but also with higher-order cognitive function. Tanaka-Koshiyama et al. (2020) evaluated resting-state EEG in patients with SCZ, and the results of their study revealed abnormally elevated EEG power in the gamma band in patients with SCZ. Therefore, this study focuses on the brain activity of schizophrenic rats in the gamma band.

Based on the above research, we established a systematic evaluation framework for brain activity status at the cortical level in schizophrenic rats using analysis method of complexity and connectivity. We collected ECoG in the frontal, parietal and occipital regions of schizophrenic and healthy rats at rest. PLZC, PE, coherence, MI and PTE were then used to analyze the brain activity state of schizophrenic rats. We hope that our work can provide a reference for research focusing on the pathological mechanisms of SCZ.

## 2 Materials and methods

### 2.1 Experimental animals

Ten healthy adult male SD rats were selected, weighing 280–300 g, Specific Pathogen Free (SPF) grade, provided by Henan Skobes Biotechnology Co., LTD. The feeding environment temperature was maintained at 25°C ± 1°C, humidity was maintained at 50∼60%, adequate water and food were given, and light and dark environment were alternated in a 12-h cycle. All procedures were approved by Xinxiang Medical University’s Animal Ethics Society (XXLL-20211015) and carried out in accordance with international experimental animal use ethics standards throughout the experiment.

### 2.2 Electrode implantation

In this paper, 10 silver wires with a diameter of 0.08 mm were used as electrode wires to prepare ECoG electrodes. The impedance of the ECoG electrodes was also tested to ensure that a high quality ECoG signal could be acquired. Channels with impedance values < 10kOhm are considered energized, otherwise they are not.

**FIGURE 1:**
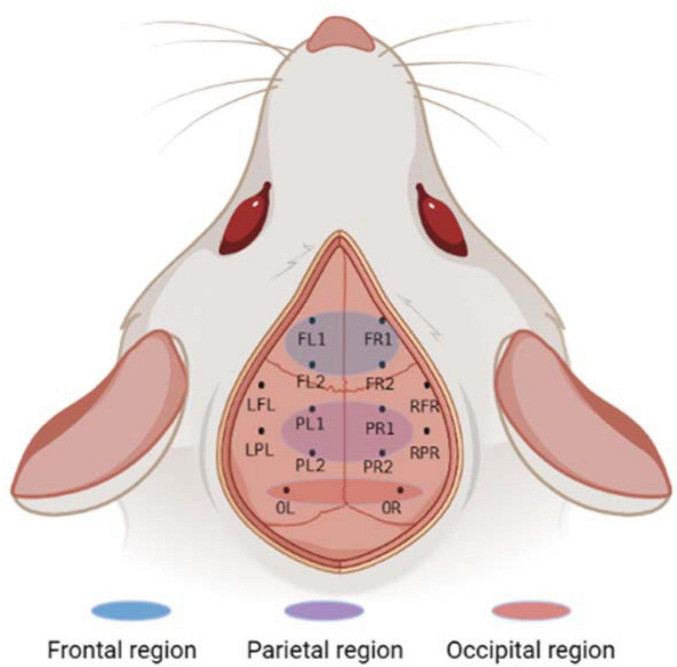
Electrode location and brain region description. The whole brain contains a total of 10 channels, of which the frontal and parietal regions have 4 channels each, the occipital region has 2 channels, and the remaining 4 channels are not in the above regions. Created with BioRender.com.

Instruments used during the procedure were rinsed with saline and placed in a sterile environment for backup. The experimental rats should be fasted for 12 h before surgery to prevent problems. After deep isoflurane (Oxygen flow rate 0.7 L/min, induction concentration 3%) anesthesia, the heads of the experimental rats was fixed to a stereoscope using an ear stick and adjusted so that the head was in a horizontal position. The surgical site was sterilized using 75% alcohol and the eyes of the experimental rats were covered with erythromycin ointment. The scalp and outer surface periosteum of the skull are removed and the fontanel is positioned. In this paper, 10 cranial nails with a diameter of 0.8 mm were used as active electrodes, and their positional coordinates relative to the fontanel are shown in Figure 1 and Table 1. In this paper, we analyzed ECoG signals in brain regions of frontal (4 electrodes: FL1, FR1, FL2, FR2), parietal (4 electrodes: PL1, PR1, PL2, PR2), and occipital (2 electrodes: OL, OR) region. The cranial nail was fixed to the head of the experimental rat according to the position of the electrode coordinates. So that the skull nail touches the dura, but does not squeeze the brain. Wrap the electrode wire of the corresponding channel around the corresponding skull nail and use dental cement to secure the electrode. After surgery, the experimental rats were kept in a single cage for seven days and injected intraperitoneally with penicillin for three days to prevent infection.

**TABLE 1:** The position of the 10 electrodes relative to the fontanel (positive values of X and Y indicate the right and front, respectively) (mm).

Location name	X	Y
FL1	−1.5	4.5
FR1	1.5	4.5
FL2	−1.5	1.5
FR2	1.5	1.5
PL1	−1.5	−1.5
PR1	1.5	−1.5
PL2	−1.5	−4.5
PR2	1.5	−4.5
OL	−3	−7
OR	3	−7

Ten screws with a diameter of 0.8 mm are used as electrodes, and their position coordinates relative to the anterior fontanel are shown in Figure 1 and Table 1. The reference electrodes are set in the cerebellum and fixed with dental cement. After the surgery, the rats were kept in a single cage for seven days, and penicillin was injected intraperitoneally for three consecutive days to prevent infection. We analyzed frontal cortical region (4 electrodes: FL1, FR1, FL2, FR2), parietal cortical region (4 electrodes: PL1, PR1, PL2, PR2), and occipital cortical region (2 electrodes: OL, OR) (see Figure 1).

### 2.3 ECoG recording

The ECoG signal acquisition device was Cerebus 128-channel neural signal acquisition system (purchased from Black Rock, USA). To improve the signal quality, the ECoG recording process was performed in a shielded room. The sampling frequency was 1,000 Hz, and the experimental rats were placed in the shielded room for 3 days before the experiment to adapt to the shielded room environment. After one week of postoperative recovery, the ECoG signals were collected from the rats. The rats were placed in the shielded room, and the Headstage was connected to the electrode connector in the awake and stationary state, and the ECoG signals were collected from the rats after the baseline was stable, and the collected ECoG signals were stored as the data of “normal rats.” The collected ECoG signal was stored as “normal rat” data. The rat model of SCZ was prepared by intraperitoneal injection of MK801 at a dose of 0.5 mg/kg, and waited for 30 min for the drug to take full effect. The ECoG signals collected were stored as “schizophrenic rat” data. The collection time was 10 s, and each rat was collected 8 times for 5 consecutive days.

### 2.4 Data pre-processing

We pre-processed the acquired ECoG signals offline in MATLAB (version 2019a, MathWorks Inc., USA). In this study, we analyzed ECoG signals in the 55–95 Hz band. First, we reduced the sampling frequency from 1,000 Hz to 256 Hz. we used a trap filter to filter the 50 Hz IF interference as well as the 100 Hz harmonics. ECoG signals with large motion artifacts were excluded by visual inspection. Finally, a band-pass filter is used to extract the ECoG signal in the 55–95 Hz band.

### 2.5 Complexity analysis

Several nonlinear dynamics algorithms have been applied to EEG analysis. These algorithms demonstrate the nonlinear characteristics of EEG signals at different levels. Among the EEG analysis methods, especially “complexity” and “entropy” are most widely used. Complexity can reflect the randomness of the signal in the time series. Entropy can be used to characterize the capacity of information. Symbolic dynamics-based measurements have the advantages of high immunity to interference and low computational complexity compared to traditional time-domain methods. Therefore, PLZC and PE are used to capture the characteristics of neural electrical activity in this paper.

#### 2.5.1 Permutation entropy

Permutation entropy (PE) is a quantitative complexity algorithm used to study the local structure of dynamic time series. It converts a given time series into a series of ordered patterns, each of which describes the sequential relationship between current and equidistant past values at a given time (Bandt and Pompe, 2002). Compared with other algorithms, PE has the characteristics of simplicity, strong anti-noise ability and low computational complexity (Li et al., 2007).

First, the given time series{*x*(*i*) : *i* ≤ 1 ≤ *N*}is reconstructed:


$$X_{i}=\{x\left(i\right),x\left(i+\pi ,\ldots ,x\left(i+\left(m-1\right)\tau \right)\right\}$$

(1) $$\begin{array}{c} i=1,2,\ldots ,N-\left(m-1\right)\,\tau \end{array}$$

In Equation 1, τ represents the time delay and *m* represents the embedding dimension.

Then the *X_i_* sequence according to Equation 2 is incrementally sorted:


(2) $$\{\,x\,\left(i+(j_{1}-1)\,\tau \right)\leq x\,\left(i+(j_{2}-1)\,\tau \right)$$

$$\leq \ldots \leq x\left(i+(j_{m}-1)\tau \right)\}$$

Therefore, there are m! permutations in the m dimension, which means that each vector *X_i_* is mapped to one of the m! permutations.

The probability of *p_j_* in Equation 3 occurring in sort j is:


(3) $$\begin{array}{c} p_{j}=\frac{n_{j}}{\sum_{j}^{m}n_{j}} \end{array}$$

*n_j_* represents the number of occurrences of the j sort.

The permutation entropy of the time series *x*(*i*) in Equation 4: *i* ≤ 1 ≤*N* is


(4) $$\begin{array}{c} H_{x}\,\left(m\right)=-\sum_{j=1}^{m!}p_{j}\,l\,n\,p_{j} \end{array}$$

When the time series is random, *H*_*x*_(*m*) reaches the maximum value ln(m!); When the time series is an ordered sequence, *H*_*x*_(*m*) tends to 0.

For ease of representation, *H*_*x*_(*m*) is usually normalized by dividing it by an ln(m!). From Equation 5, the permutation entropy of the time series x(i):


(5) $$\begin{array}{c} P\,E=\frac{H_{x}\,\left(m\right)}{\ln\left(m!\right)} \end{array}$$

#### 2.5.2 Permutation Lempel-Ziv complexity

Permutation Lempel-Ziv complexity (PLZC) is proposed based on Lempel-Ziv complexity combined with sorting method (Liu et al., 2023), In the sorting process, embedding dimension m and delay time τ are the two most important parameters. The calculation of PLZC can be realized in the following 8 steps:

The first step is to use a sorting algorithm to convert the EEG signal into a finite digital sequence {x(n)}. Through this step, the signal will be represented as no more than m! symbols representing the sorting mode. The second step is to initialize the measurement values of PLZC. Make S and Q represent the first and second symbols in {x(n)}, respectively, and make the complexity c(n) = 1. The third step is to integrate S and Q into SQ, removing the end characters of the SQ sequence to form SQv. Fourth, determine whether Q has reached the end of the character sequence. If it has already arrived, the algorithm result will be normalized. Fifth, take a subsequence of SQv. Place all subsequences in a table named SQv^sub^ if Q belongs to SQv^sub^, then proceed to step 6. Otherwise, Q is a new sequence and we proceed to step 7. Step 6, add the next character to update Q and return to step 3. Step 7, set S = SQ and assign Q to the next character in the numerical sequence {x(n)}. At the same time, the complexity c(n) is increased by 1. Step 8, the complexity c(n) obtained at this point represents the number of different patterns in the original number sequence {x(n)}. From Equation 6, the total number of subsequences has an upper bound (Borowska, 2021) L(n). From Equation 7, PLZC can be expressed as:


(6) $$\begin{array}{c} \text{L}\,\left(\text{n}\right)=\text{c}\,\left(\text{n}\right)\,{\text{log}}_{m!}\,\left[\text{c}\,\left(\text{n}\right)\right]+1 \end{array}$$

(7) $$\begin{array}{c} \text{PLZC}=\frac{\text{c}\left(\text{n}\right)\left({\text{log}}_{m!}\left[\text{c}\left(\text{n}\right)\right)+1\right)}{\text{n}} \end{array}$$

### 2.6 Connectivity analysis

To assess information interactions between brain regions in schizophrenic rats. In this paper, we analyze the EEG connectivity evaluation metrics from time domain, frequency domain and directed information flow, respectively. In this paper, connectivity measurements including coherence, MI and PTE between different channels are calculated for connectivity studies.

#### 2.6.1 Coherence

Coherence can measure the linear relationship between two signals in a specific frequency band or frequency point. Assuming that X (t) and Y (t), respectively, represent the EEG signals of electrode (or brain area) X and Y (Niso et al., 2013). Firstly, the frequency domain conversion method is used to convert the time-domain signals X(t) and Y(t) to the frequency domain. Then, for each frequency f, estimate its respective spectral power density *S*_*xx*_(*f*) and *S*_*yy*_(*f*) and their cross-power density *S*_*xy*_(*f*). In view of the above, the coherence function between them can be calculated using Equation 8 (coherency function)*K*_*xy*_(*f*):


(8) $$\begin{array}{c} K_{x\,y}\,\left(f\right)=\frac{S_{x\,y}\,\left(f\right)}{\sqrt{S_{x\,x}\,\left(f\right)\,S_{y\,y}\,\left(f\right)}} \end{array}$$

Finally, calculate the coherence value at frequency f using Equation 9:


(9) $$\begin{array}{c} C\,O\,H_{x\,y}\,\left(f\right)={\left|K_{x\,y}\,\left(f\right)\right|}^{2} \end{array}$$

The range of values for coherent indicators is 0∼1. *COH*_*xy*_(*f*) 0 means that there is no linear dependence between X(t) and Y(t) at frequency f. The larger the coherence value, the stronger the statistical dependency between the two signals; The reverse is also true.

#### 2.6.2 Mutual information

In information theory, mutual information is an extension of information entropy, commonly used to measure the correlation between two random variables (Melia et al., 2015). From Equations 10–12, define the entropy of two time series as H(x) and H(y), and the joint entropy between them as H (x, y):


(10) $$\begin{array}{c} H\,\left(x\right)=-\sum_{x=X}p_{x}\,\left(x\right)\,l\,o\,g\,p_{x}\,\left(x\right) \end{array}$$

(11) $$\begin{array}{c} H\,\left(y\right)=-\sum_{y=Y}p_{y}\,\left(y\right)\,\log p_{y}\,\left(y\right) \end{array}$$

(12) $$\begin{array}{c} H\,\left(X,Y\right)=-\sum_{x=X,y=Y}p\,\left(x,y\right)\,\log p\,\left(x,y\right) \end{array}$$

P (x, y) is the joint probability density of X and Y. From Equation 13, mutual information is defined as:


(13) $$\begin{array}{c} I\,\left(X,Y\right)=H\,\left(X\right)+H\,\left(Y\right)-H\,\left(X,Y\right) \end{array}$$

#### 2.6.3 Phase transfer entropy

Phase Transfer Entropy (Lobier et al., 2014; Zhu et al., 2023) (PTE) has high computational efficiency, can reliably detect directional interactions between signals, is robust to noise and linear mixing, and is suitable for large-scale directional connectivity analysis. At the same time, due to the asymmetry of PTE, PTE can be used to represent the direction of information flow between neuron groups.

The instantaneous phase time series of signals X and Y are defined as θ_*x*_(*t*) and θ_*y*_(*t*), which are obtained by the Hilbert transformation of the two signal time series. The PTE from X to Y between signals X and Y is shown in Equation 14:


(14) $$\begin{array}{c} P\,T\,E_{x\,y}=H\,\left(\theta_{y}\,\left(t\right),\theta_{y}\,\left(t^{\prime }\right)\right)+H\,\left(\theta_{y}\,\left(t^{\prime }\right),\theta_{x}\,\left(t^{\prime }\right)\right)\\ -H\,\left(\theta_{y}\,\left(t^{\prime }\right)\right)-H\,\left(\theta_{y}\,\left(t\right),\theta_{y}\,\left(t^{\prime }\right),\theta_{x}\,\left(t^{\prime }\right)\right) \end{array}$$

### 2.7 Statistical analysis

We statistically analyzed the experimental results using SPSS 19.0 software. For each set of data, normality was tested using the Shapiro–Wilk test, and variance chi-square was examined using Levene’s test. If the data satisfied the normal distribution and the variance was homogeneous, the paired-samples *t*-test was used; if the data did not satisfy the normal distribution or the variance was not homogeneous, the Wilcoxon rank-sum test was used. *P* < 0.05 indicated that there was a statistically significant difference between the two groups of data, and *P* < 0.001 indicated that there was a significant statistically significant difference between the two groups of data. In the figure, *P* < 0.05, *P* < 0.01 and *P* < 0.001 are represented by the symbols *, **, ***.

To evaluate the relationship between complexity and functional connectivity, we used the Pearson correlation coefficients, R of between PE and coherence, R of PE and MI, R of PLZC and coherence, R of PLZC and MI.

Finally, we conducted surrogate analysis to test the significance of the estimated PTE values. The estimated phases from the Hilbert transform for electrodes from a given pair of brain areas were time-shuffled so that the predictability of one time-series from another is destroyed, and PTE analysis was repeated on this shuffled data to build a distribution of surrogate PTE values against which the observed PTE was tested (*P* < 0.05).

## 3 Results

### 3.1 The changes of neuronal activity in schizophrenic rats

**FIGURE 2:**
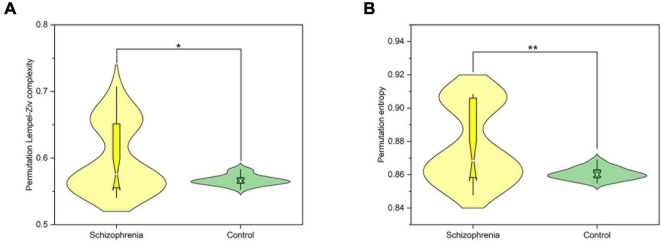
The nonlinear dynamic characteristics of the whole brain. **(A)** The average PLZC values of the whole brain. **(B)** The average PE values of the whole brain. *P* < 0.05 and *P* < 0.01 are represented by the symbols *, **.

In order to further analyze the changes of nonlinear dynamic characteristics in the whole brain of the experimental rats, the Wilcoxon rank sum test was performed on the PLZC and PE values of the two groups of rats in this paper, and the results are shown in Figure 2. The PLZC values of schizophrenic rats were statistically significantly higher than those of normal rats (*Z* = 2.325, *P* = 0.02) (Figure 2A). The PE values were statistically significantly higher in schizophrenic rats than in normal rats (*Z* = 3.199, *P* = 0.001) (Figure 2B).

**FIGURE 3:**
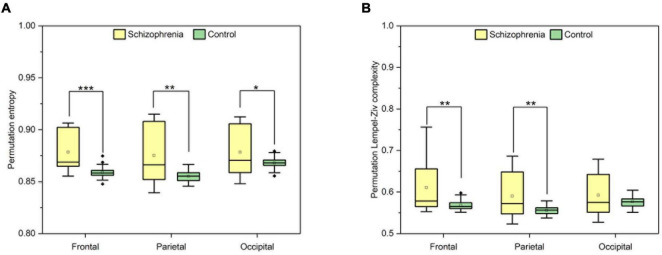
The complexity of frontal, parietal and occipital regions. **(A)** The value of PLZC of frontal, parietal and occipital regions. **(B)** The value of PE of frontal, parietal and occipital regions. Boxplots show the median and the four quartiles, where outliers beyond two interquartile intervals from the median are shown as individual dots. *P* < 0.05, *P* < 0.01 and *P* < 0.001 are represented by the symbols *, **, ***.

Finally, in order to elucidate the differences among different brain regions in the SCZ rat brain, we analyzed the neural activities within the frontal, occipital and parietal brain regions. Still using the Wilcoxon rank sum test, all three brain regions were found to be statistically significant by the calculation and the results are shown in Figure 3. Compared with normal rats, SCZ rats showed similar changes in PLZC and PE values in the three brain regions. Wilcoxon rank sum test showed that the PE values of SCZ rats were lower than those of normal rats in all three brain regions (frontal: *Z* = 4.785, *P* < 0.001; parietal: *Z* = 3.145, *P* = 0.002; occipital: *Z* = 2.124, *P* = 0.034) (See Figure 3A). PLZC values in the frontal and parietal lobes of SCZ rats were lower than those of normal rats (frontal: *Z* = 3.098, *P* = 0.002; parietal: *Z* = 2.917, *P* = 0.004) (See Figure 3B).

### 3.2 Changes of information integration between cortical regions in schizophrenic rats

**FIGURE 4:**
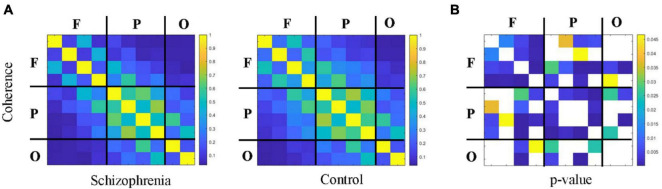
The change in coherence within 55∼95 Hz frequency band. **(A)** Coherence matrix of one rat with black straight lines dividing the 10 channels into the frontal, parietal, and occipital regions (denote as F, P, O, respectively). **(B)** The statistical results of adjacency matrix.

**FIGURE 5:**
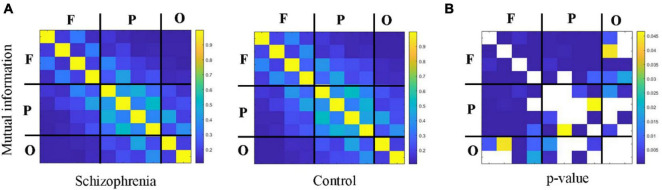
The change in MI within 55∼95 Hz frequency band. **(A)** MI matrix of one rat with black straight lines dividing the 10 channels into the frontal, parietal, and occipital regions (denote as F, P, O, respectively). **(B)** The statistical results of adjacency matrix.

Figures 4, 5 showed the adjacency matrix of coherence and MI in the high gamma frequency band for two groups of rats. The values in the adjacency matrix show a symmetrical distribution and the matrix is divided into nine parts using a black line, which is used to distinguish the three brain regions. The results showed that SCZ rats had lower mutual information and coherence values than normal rats in all three brain regions. In SCZ rats, mutual information and coherence values were higher between frontal and parietal channels and lower among occipital channels.

**FIGURE 6:**
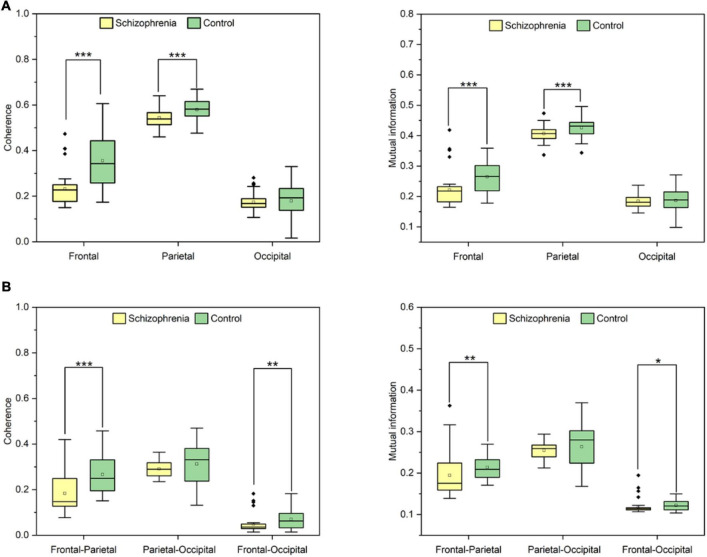
**(A)** The results of coherence and mutual information in brain regions. **(B)** The statistical results of coherence and mutual information in frontal-occipital, frontal-parietal and parietal-occipital regions. *P* < 0.05, *P* < 0.01 and *P* < 0.001 are represented by the symbols *, **, ***.

First, we analyzed connectivity in the rat brain region. Linear and nonlinear connectivity within the frontal, parietal, and occipital brain regions are represented in Figure 6A. Wilcoxon rank sum test and paired samples *t*-test showed that SCZ rats had statistically significant lower values of coherence and mutual information within the frontal, parietal, and occipital brain regions than the normal rats in the resting state. Coherence was statistically significant between the two groups (frontal: *Z* = −4.422, *P* < 0.001; occipital: *t* = −3.937, *P* < 0.001). Mutual information was statistically significant between the two groups (frontal: *Z* = −3.459, *P* < 0.001; occipital: *t* = −3.334, *P* < 0.001).

Second, we analyzed the connectivity between brain regions in rats. Statistical differences in changes in linear and nonlinear connectivity between brain regions are shown in Figure 6B. Wilcoxon rank sum test showed that linear and nonlinear connectivity between frontal-parietal lobes were statistically significantly lower in SCZ rats than in normal rats in the resting state. The linear connectivity between frontal-parietal lobes was statistically significant between the two groups (*Z* = −3.723, *P* < 0.001), and that between frontal-occipital lobes was statistically significant between the two groups (*Z* = −2.775, *P* = 0.006). Non-linear connectivity between frontal-parietal lobes was statistically significant between the two groups (*Z* = −2.661, *P* = 0.008) and between frontal-occipital lobes (*Z* = −2.245, *P* = 0.025).

**FIGURE 7:**
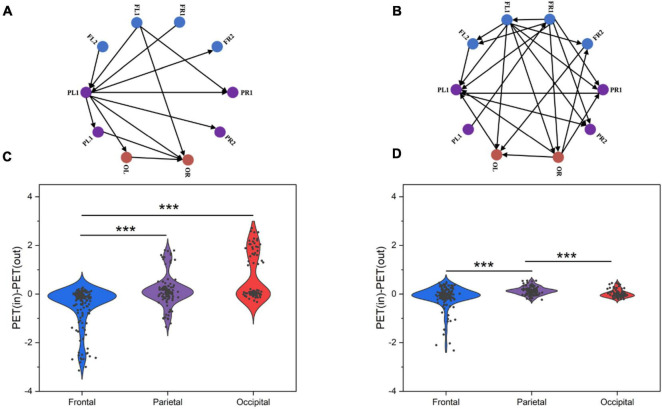
Information flow as measured by PTE for chosen cortical networks. **(A)** Information flow in normal rat PTE network. **(B)** Information flow in SCZ rat PTE network. **(C)** Outflow from frontal, parietal and occipital lobes of control rats. **(D)** Outflow from frontal, parietal and occipital lobes of SCZ rats. *P* < 0.001 is represented by the symbols ***.

To further visualize the pattern of causal interactions, we calculated causal associations between the frontal, parietal, and occipital lobes in resting state between normal and SCZ rats. SCZ rats had more and denser causal interactions among the three brain regions than normal rats (Figures 7A, B). Next, we tried to quantify the net outflow and inflow from each node of the three brain regions. Specifically, we calculated the outflow from each region, defined as the sum of the PTE from electrodes in one brain region to electrodes in the other two brain regions, and the opposite inflow. The difference between inflow and outflow is used to determine the inflow hub. This analysis determined that the parietal and occipital lobes were the strongest inflow areas in normal rats (Figure 7C). However, in SCZ rats, it is not clear which brain region is the center of information exchange (Figure 7D).

### 3.3 The correlation between connectivity and complexity

**FIGURE 8:**
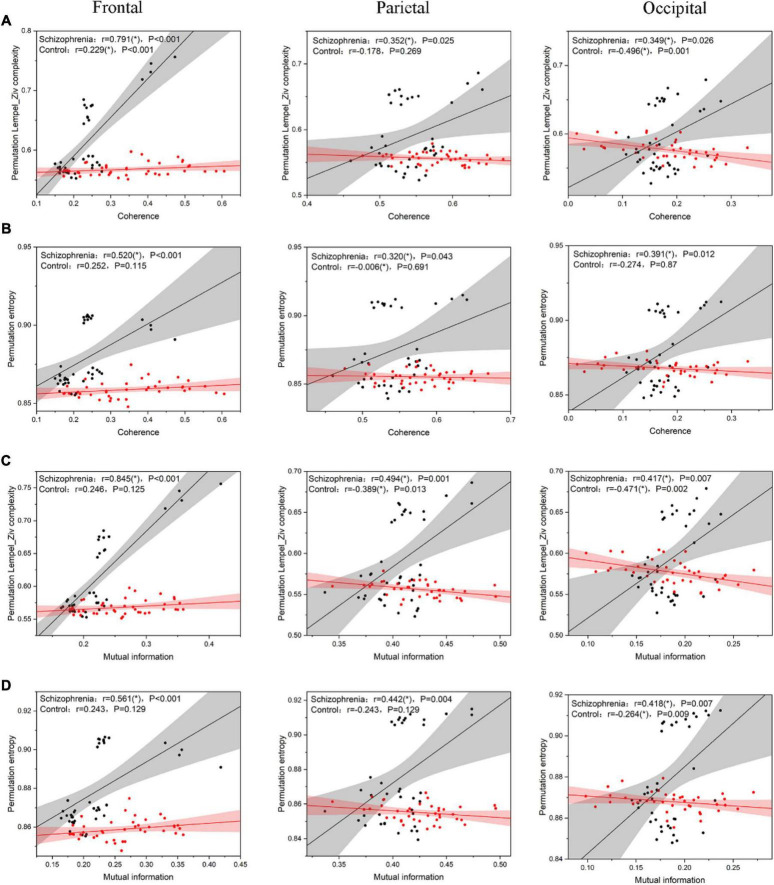
the scatter plot between complexity and connectivity indicators. **(A)** The results of Pearson correlation analysis between PLZC and coherence. **(B)** The results of Pearson correlation analysis between PE and coherence. **(C)** The results of Pearson correlation analysis between PLZC and mutual information. **(D)** The results of Pearson correlation analysis between PE and mutual information. The solid line in the figure is the linear regression line (black is the linear regression line of SCZ rats, red is the linear regression line of normal rats), and *r* is the Pearson correlation coefficient [*r* value satisfying *P* < 0.05 is represented by (*)].

According to Ferriston’s study, SCZ is a functional disconnection and this disconnection may be associated with higher complexity. Therefore, in order to assess the relationship between complexity and functional connectivity of brain regions, we analyzed the Pearson correlation coefficients between complexity and functional connectivity indexes of brain regions in SCZ rats and normal rats, and the results were shown in Figure 8.

The complexity of the three brain regions of the SCZ rats and their functional connectivity were highly and positively correlated (PLZC-coherence (Figure 8A): frontal: *r* = 0.791, *P* < 0.001; parietal: *r* = 0.352, *P* = 0.025; occipital: *r* = 0.349, *P* = 0.026. PE-coherence (Figure 8B): frontal: *r* = 0.520, *P* < 0.001; parietal: *r* = 0.320, *P* = 0.043; occipital: *r* = 0.391, *P* = 0.012. PLZC-MI (Figure 8C): frontal: *r* = 0.845, *P* < 0.001; parietal: *r* = 0.494, *P* = 0.001; occipital: *r* = 0.417, *P* = 0.007. PE-MI (Figure 8D): frontal: *r* = 0.561, *P* < 0.001; parietal: *r* = 0.442, *P* = 0.004; occipital: *r* = 0.418, *P* = 0.007). The complexity of the three brain regions in normal rats was negatively or less positively correlated with their functional connectivity (PLZC-coherence (Figure 8A): frontal: *r* = 0.229, *P* < 0.001; parietal: *r* = −0.178, *P* = 0.269; occipital: *r* = −0.496, *P* = 0.001. PE-coherence (Figure 8B): frontal: *r* = 0.252, *P* = 0.115; parietal: *r* = −0.006, *P* = 0.691; occipital: *r* = −0.274, *P* = 0.870. PLZC-MI (Figure 8C): frontal: *r* = 0.246, *P* = 0.125; parietal: *r* = −0.389, *P* = 0.013; occipital: *r* = −0.471, *P* = 0.002. PE-MI (Figure 8D): frontal: *r* = 0.243, *P* = 0.129; parietal: *r* = −0.243, *P* = 0.129; occipital: *r* = −0.264, *P* = 0.009).

## 4 Discussion

In this study, we systematically studied the state of brain activity in SCZ at the cortical level. The study found that the SCZ rat model was similar to schizophrenic patients in terms of information interaction between brain regions, complexity, and brain networks. This could provide new understanding of neural network processes in healthy brains and in pathological states. The findings suggest that both complexity and connectivity-based brain networks have the potential to assess abnormal EEG activity in a rat model of SCZ.

### 4.1 Frontal and parietal regions are associated with brain dysfunction in SCZ rats

In the frontal and parietal region, we found connectivity and nonlinear dynamics indicators that can distinguish between schizophrenic rats and normal rats. However, features of the occipital brain region do not possess this comprehensive ability, and these results all confirm that the frontal-parietal brain region is involved in SCZ dysfunction. The complexity and connectivity abnormalities of EEG in patients with SCZ, especially in the frontal and parietal regions of the brain (Mathalon and Ford, 2008). Previous studies have reported that functional magnetic resonance imaging (fMRI) results of patients with SCZ show abnormalities in brain structure and morphology (Highley et al., 2018). Recent studies have shown that brain electrical activity in the frontal and parietal lobes is thought to play a key role in human working memory (Kuo and Van Petten, 2008). Jeong et al. (1998) reported that patients with SCZ showed significant abnormalities in the correlation dimension of the left frontal lobe. Akar et al. (2016) found significant abnormalities in the complexity of frontal and parietal regions of the brain in patients with SCZ. In the present study, our findings showed that the PE and PLZC values in the frontal and parietal lobes of the schizophrenic rat model were significantly abnormal, whereas those in the occipital lobe were not, which is consistent with the results of the aforementioned literature. Deserno et al. (2013) found reduced prefrontal-parietal connectivity in patients with SCZ. Cui et al. (2016) found that exhibiting low connectivity within frontal and parietal networks. In addition, some researchers have shown that this disease is believed to be closely related to the dysfunction of the functional structure (Pu et al., 2016; M’barek et al., 2022) and disconnection (Williams et al., 2007) of the frontal region. The above EEG-based results can explain why we observed abnormal connectivity in the frontal and parietal lobes in a rat model of SCZ. Previous findings have shown that patients with SCZ have structural abnormalities in the parietal brain region (Dorph-Petersen and Lewis, 2017; Pan et al., 2017), Frontal–parietal connectivity disorder is an important pathology of SCZ (Son et al., 2017; Xiang Q. et al., 2019). SCZ patients have dysfunction and abnormal connectivity, especially in the functional structure of the frontal lobe (Mathalon and Ford, 2008). These results further support that abnormal EEG activity in the frontal and parietal lobes is associated with brain dysfunction in a rat model of SCZ.

### 4.2 Complexity index is a powerful tool to identify SCZ

Ferriston’s disconnection syndrome hypothesis has demonstrated the presence of complexity abnormalities in SCZ patients, i.e., abnormal connections between neural systems due to dissociation or splitting between brain regions (Sporns et al., 2000). Recent findings have shown that MEG signaling is significantly more complex in patients with SCZ than in healthy controls (Bai et al., 2022). This may support our findings. The findings of the present study showed that the PE and PLZC values of the rat model of SCZ were significantly higher than those of the control group. Xiang J. et al. (2019) suggested that the EEG signals of schizophrenic patients have a higher degree of irregularity or variability, suggesting potentially disorganized, irregular neuronal spiking activity. One possible explanation is that this increase in complexity and entropy can be understood as an increase in the number of complex interconnected and simultaneously active neural components (Bai et al., 2015). For example, (Zhao et al., 2013) found that schizophrenic patients had more active and excitable brain activity, a greater probability of generating new patterns of EEG signals, and higher EEG complexity compared to normal controls. Under such conditions of increased neural complexity, brain activity operates in an asynchronous parallel distribution pattern, which may lead to self-organization and dynamic instability (Arshavsky, 2016). Thus, nonlinear neurodynamic features are sensitive to neuronal activity and brain states, providing an important avenue for exploring the mechanisms of cognitive dysfunction in the SCZ (Xiang J. et al., 2019).

### 4.3 Reduced information coupling ability between brain regions in rats with SCZ

In the literature on SCZ, conflicting results have been reported on brain network function, and the differences in research results are mainly due to differences in different methods. For example, Hummer used the mean and variance of correlation coefficients across brain regions to define network connectivity, and the results of his study found that whole-brain functional connectivity was diminished in schizophrenics compared to controls (Hummer et al., 2020). Yin et al. (2017) used MI to construct a functional brain network with fewer informational interactions in SCZ patients compared to normal controls. Yeum and Kang (2018) found reduced coherence between brain regions in schizophrenic patients. Different measures of brain network connectivity are used to quantify the ability of information coupling in brain networks (Abazid et al., 2021). This study uses spectral coherence and mutual information methods to evaluate the functional connectivity of ECoG signals in schizophrenic rats, and analyzes the electrophysiological changes of the brain network in linear and nonlinear coupling. The trends of the two are consistent, with a decrease in linear interaction and a decrease in nonlinear coupling. One possible explanation is that this decline in linear connectivity indicates damage to cortical connections or a separation between cortical and subcortical structures, and a weakening of nonlinear coupling is associated with reduced functional connectivity between cortical structures (Alonso et al., 2010).

### 4.4 Causal structural abnormalities in directional information flow between different neural oscillatory activities in SCZ rats

For PET results, causal interactions between brain regions in SCZ rats were more complex. But the intensity of information interaction between brain regions decreased. This is consistent with the results of functional connectivity and complexity analysis in this paper. Rajpal et al. (2022) found that the diversity of EEG signals increased in patients with SCZ, and the causal relationship between brain regions was close. The reason for this phenomenon may be that abnormal activity of the dopamine system in SCZ rats may be associated with the appearance of symptoms such as hallucinations and delusions. Abnormalities in this neurotransmitter system may affect the functional activities of brain regions, making causal interactions between brain regions more intimate. This may imply that brain activity in the SCZ rat model is under tension in the resting state (Barnett et al., 2020).

### 4.5 The tighter and more complex the connections between brain regions in SCZ rats

Fernández et al. (2013) proposed a functional disconnection syndrome in SCZ, suggesting that this disconnection is associated with higher and higher complexity. The pathology of SCZ leads to cortical disconnection, which leads to abnormal interactions between different regions, resulting in dysfunctional connectivity and complexity abnormalities (Sporns et al., 2000). The findings suggest that functional connectivity of brain regions is positively correlated with complexity in schizophrenic rats. Lynall et al. (2010) found that the strength of functional connectivity was significantly reduced in patients with SCZ, while the diversity of functional connectivity was increased. In the process of reducing the effective coupling strength of the fully synchronized state, Pikovsky et al. (2002) argued that nonlinear coupled oscillations can appear as chaotic states, which can increase complexity. The positive correlation observed in this study can be used to explain this mechanism. The above findings suggest that the combination of complexity and functional connectivity may reflect the complex pathologic processes of SCZ.

## 5 Conclusion

In this study, we found that the connectivity of brain regions in schizophrenic rats increases with increasing complexity. At the cortical level, the complexity of brain regions in schizophrenic rats decreased, the ability of information integration between cortical brain regions decreased, and the information exchange of brain networks was blocked. We also found causal structural abnormalities in the directional information flow between different neural oscillatory activities in schizophrenic rats. It is concluded that complexity and connectivity are valid indicators for diagnosing SCZ. In addition, our study fills the gap between spike and EEG in understanding the pathological mechanisms of SCZ at the cortical level.

However, there are still some limitations that should be considered in the future. First, subsequent studies could increase the number of leads as well as the task status. This helps to analyze the link between the function of different brain regions and cognitive deficits in the SCZ. Second, subsequent studies could increase the length of the acquired signal. This helps to understand the dynamic information interactions between brain regions.

## Data availability statement

The datasets used and analyzed in the current study are available from the corresponding author upon reasonable request.

## Ethics statement

The animal study was approved by the Ethics Committee of Xinxiang Medical College (XXLL-20211015). The study was conducted in accordance with the local legislation and institutional requirements.

## Author contributions

ZZ: Conceptualization, Data curation, Formal analysis, Funding acquisition, Investigation, Methodology, Project administration, Resources, Software, Supervision, Validation, Visualization, Writing – original draft, Writing – review & editing. YF: Conceptualization, Data curation, Formal analysis, Funding acquisition, Investigation, Methodology, Project administration, Resources, Software, Supervision, Validation, Visualization, Writing – original draft, Writing – review & editing. JW: Conceptualization, Data curation, Formal analysis, Funding acquisition, Investigation, Methodology, Project administration, Resources, Software, Supervision, Validation, Visualization, Writing – original draft, Writing – review & editing. TT: Conceptualization, Data curation, Formal analysis, Funding acquisition, Investigation, Methodology, Project administration, Resources, Software, Supervision, Validation, Visualization, Writing – original draft, Writing – review & editing. RL: Conceptualization, Data curation, Formal analysis, Funding acquisition, Investigation, Methodology, Project administration, Resources, Software, Supervision, Validation, Visualization, Writing – original draft, Writing – review & editing. MenghW: Conceptualization, Data curation, Formal analysis, Funding acquisition, Investigation, Methodology, Project administration, Resources, Software, Supervision, Validation, Visualization, Writing – original draft, Writing – review & editing. HH: Conceptualization, Data curation, Formal analysis, Funding acquisition, Investigation, Methodology, Project administration, Resources, Software, Supervision, Validation, Visualization, Writing – original draft, Writing – review & editing. MengkW: Conceptualization, Data curation, Formal analysis, Funding acquisition, Investigation, Methodology, Project administration, Resources, Software, Supervision, Validation, Visualization, Writing – original draft, Writing – review & editing. PC: Conceptualization, Data curation, Formal analysis, Funding acquisition, Investigation, Methodology, Project administration, Resources, Software, Supervision, Validation, Visualization, Writing – original draft, Writing – review & editing. XG: Conceptualization, Data curation, Formal analysis, Funding acquisition, Investigation, Methodology, Project administration, Resources, Software, Supervision, Validation, Visualization, Writing – original draft, Writing – review & editing. YW: Conceptualization, Data curation, Formal analysis, Funding acquisition, Investigation, Methodology, Project administration, Resources, Software, Supervision, Validation, Visualization, Writing – original draft, Writing – review & editing. ChaW: Conceptualization, Data curation, Formal analysis, Funding acquisition, Investigation, Methodology, Project administration, Resources, Software, Supervision, Validation, Visualization, Writing – original draft, Writing – review & editing. ZG: Conceptualization, Data curation, Formal analysis, Funding acquisition, Investigation, Methodology, Project administration, Resources, Software, Supervision, Validation, Visualization, Writing – original draft, Writing – review & editing. WJ: Conceptualization, Data curation, Formal analysis, Funding acquisition, Investigation, Methodology, Project administration, Resources, Software, Supervision, Validation, Visualization, Writing – original draft, Writing – review & editing. XZ: Conceptualization, Data curation, Formal analysis, Funding acquisition, Investigation, Methodology, Project administration, Resources, Software, Supervision, Validation, Visualization, Writing – original draft, Writing – review & editing. ML: Conceptualization, Data curation, Formal analysis, Funding acquisition, Investigation, Methodology, Project administration, Resources, Software, Supervision, Validation, Visualization, Writing – original draft, Writing – review & editing. ChoW: Conceptualization, Data curation, Formal analysis, Funding acquisition, Investigation, Methodology, Project administration, Resources, Software, Supervision, Validation, Visualization, Writing – original draft, Writing – review & editing. YY: Conceptualization, Data curation, Formal analysis, Funding acquisition, Investigation, Methodology, Project administration, Resources, Software, Supervision, Validation, Visualization, Writing – original draft, Writing – review & editing. TP: Writing – review & editing.
